# Full-polarization-locked vortex beam generator with time-varying characteristics

**DOI:** 10.1515/nanoph-2023-0947

**Published:** 2024-02-09

**Authors:** Lixin Jiang, Yongfeng Li, Hao Yang, Shuang Liang, Lin Zheng, Zhe Qin, Zhibiao Zhu, Hongya Chen, Jiafu Wang, Shaobo Qu

**Affiliations:** Shaanxi Key Laboratory of Artificially-Structured Functional Materials and Devices, Air Force Engineering University, Xi’an, Shaanxi 710051, China

**Keywords:** time-varying vortex beam, PB phase, dynamic phase, full polarization

## Abstract

Vortex beams carrying orbital angular momentum (OAM) are considered to hold significant prospects in fields such as super-resolution imaging, high-capacity communications, and quantum optics. Therefore, the techniques of vortex beam generation have attracted extensive studies, in which the development of metasurfaces brings new vigor and vitality to it. However, the generation of reconfigurable vortex beams by metasurfaces at the incidence of arbitrary polarized electromagnetic (EM) waves holds challenges. In this study, an efficient and reconfigurable strategy utilizing PB phase-modulated circularly polarized waves and dynamic phase-modulated linearly polarized waves is proposed, enabling a polarization-locked fully polarization vortex beams generator. Based on this strategy, we designed and fabricated a prototype of the vortex beam generator for full polarization, which verifies the rotating Doppler effect and generates a time-varying vortex beam. All the results have been verified by simulation and measurements. In addition, the proposed strategy can be easily extended to other frequency regions and holds potential in areas such as information encryption, biosensing, and OAM multiplexing communication.

## 1 Introduction

Angular momentum is one of the fundamental physical quantities of classical and quantum mechanics [1]. Electromagnetic (EM) waves carry spin angular momentum and orbital angular momentum (OAM) [2], with OAM in particular attracting attention for the new degrees of freedom it provides. The vortex beam carrying the OAM has a circular intensity distribution and a helical phase profile exp(*jlφ*) in the *x*–*y* plane. Since OAM patterns with different topological charges *l* are orthogonal, and *l* can take values from negative infinity to positive infinity, which exhibits great potential in expanding the channel capacity [3], [4], [5]. Besides, vortex beams are used in optical tweezers [6], [7], [8], optical imaging [9], [10], [11], super-resolution microscopy [12], [13], [14], etc. Therefore, how to generate vortex beams has become a research hotspot. Traditional methods of generating vortex beams include spiral phase plates [15], [16], [17], Q-plates [18], and computer-generated holograms [19]. However, such devices, which modulate phase by accumulating propagation distances to generate vortex beams, are bulky and limit the further advancement of integrated optics. In addition, traditional devices require high-precision fabrication and can only generate vortex beams with specific topological charges, limiting their practical applications. Recently, the spatio-temporal optical vortices carrying transverse OAM with a vortex phase in the space-time plane (*x*–*t* plane) have stirred up attention. The new degrees of freedom given to OAM by spatio-temporal vortices [20], [21], [22], [23] enable it to show great potential for space-time differentiators [24], [25], subvelocity of light and superluminal pulse propagation [26], [27], etc. However, traditional devices appear impotent for generating spacetime vortices. A range of other methods, including extreme optical pulses [28] and high-order harmonics [29], have been used to generate spatio-temporal optical vortices. Although significant achievements have been achieved in vortex beam generation, new strategies for generating vortex beams using metasurfaces or other nanophotonic structures are highly desired to miniaturize the system and achieve reconfigurability.

Metasurfaces, an artificially designed 2D material, have been highlighted as a particularly attractive method of generating vortex beams. Since the proposal of generalized Snell’s law [30] by Prof. Capasso in 2011, metasurfaces have demonstrated a strong ability to modulate the amplitude, polarization, phase, and frequency of light fields. Consequently, numerous fascinating functions have been explored using metasurfaces, ranging from holographic imaging [31], [32], [33], circular dichroism [34], [35], [36], [37], wavefront modulation, and so on. In particular, the introduction of active devices greatly enhances the functionality of the metasurface, including space-time modulation [38], [39], [40], [41], [42], dynamic reflection [43], [44], [45], [46], etc. Microstructures in the metasurface can independently or simultaneously control the dynamic and Pancharatnam–Berry (PB) phases [47], [48] to generate vortex beams. Hence, generating non-reconfigurable vortex beams [49], [50] has been extensively studied. Meanwhile, reconfigurable vortex beams can be realized by dynamically modulating the reflection phase using active devices such as varactor diodes [51] and phase-change materials [52]. For instance, Sedeh et al. [53] designed a time-modulated metasurface with a linear azimuthal frequency gradient, demonstrating the generation of optical vortices with time-varying topological charges. Further, Zhang et al. [22] realized time-varying vortex beams in the microwave band using a space-time metasurface with a field-programmable gate array (FPGA). However, most reports on the implementation of dynamic vortex beams have concentrated on single-polarized EM waves based on the dynamic phase. And the introduction of varactors remains the most comprehensive approach to dynamically modulating phase. However, the relationship between capacitance value and phase is nonlinear [54], [55]. Even small frequency shifts can cause a dramatic variation in the phase difference between the two states. Moreover, dynamic modulation of PB phases with broadband and dispersion-free is usually limited to 1-bit due to the difficulty in achieving dynamic rotation of meta-atoms. Recently, Feng et al. [56] realized reconfigurable PB phases using mechanical motors to control the rotation of each meta-atom. However, the slow response and complex control of mechanical modulation hinder its practical applications. To the best of our knowledge, there has been rarely reported about fusing PB and dynamic phases to realize reconfigurable vortex beam generators under the incidence of full polarization EM waves, which is imperative to enhance the utility of the generators.

**Figure 1:**
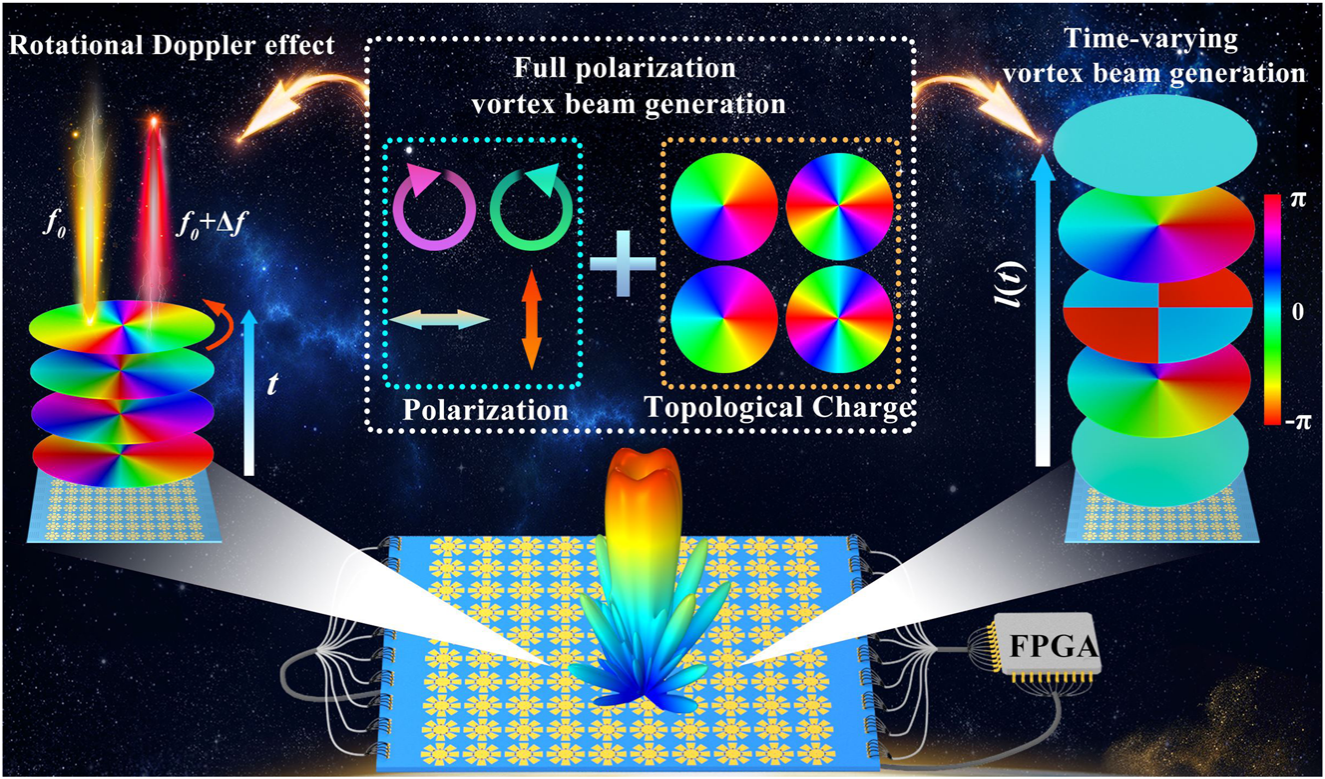
Schematic of vortex beam generator for full polarization. The whole system is composed of a metasurface and an FPGA control system. The polarization-locked vortex beams with tunable topological charge can be achieved at arbitrarily polarized wave incidence, as shown on the left. Time-varying vortex beams with time-related topological charges are generated, as shown on the right.

Here, we proposed a voltage-modulated multi-bit PB and dynamic phases switchable strategy in the microwave regime. A space-time coding metasurface containing meta-atoms with 8-fold rotational symmetry is designed to modulate the dynamic phase by changing the capacitance of the varactor diode for the linear polarized (LP) EM wave. Equivalent rotation of the meta-atoms is achieved by symmetry breaking via conducting different varactor diodes, modulating the PB phase for CP wave. In this strategy, the dynamic phase for LP wave and PB phase for CP wave can be dynamically varied and cover 2*π*, which underpins the generation of vortex beams with tunable topological charges under the incidence of full polarization EM waves. As a demonstration, a prototype operating in the C-band with a 2-bit coding phase for full polarization EM wave incidence was designed and fabricated, as shown in Figure 1. The vortex beams with topological charges of −2, −1, +1, and +2 were generated by designing the coding sequence, which was validated by simulations and measurements. Furthermore, the rotating Doppler effect [57], [58] was demonstrated, and the time-varying vortex beams were generated by space-time coding sequences. In conclusion, our strategy provides an unexplored platform for developing reconfigurable vortex beams, which can be further applied in communication encryption and probing the chirality of molecules.

## 2 Materials and theoretical design

Vortex beam generator with tunable topological charge at arbitrary polarization wave incidence requires 2*π* reflection phase coverage with polarization-locked. The most common method is to achieve 2*π* phase coverage by loading varactor diodes on the meta-atom. For LP waves, the reflected phase modulation with polarization retention can be achieved by simultaneously varying the capacitance value of all varactor diodes on the isotropic meta-atom. However, the isotropic meta-atom changes the polarization of the reflected CP wave. Therefore, an anisotropic meta-atom needs to be designed to achieve spin-locked reflection for CP waves. Here, the meta-atom is anisotropic when the varactor diodes in a single arm are turned on. And the equivalent rotation of the anisotropic meta-atom is achieved by conducting the varactor diode in different arms. Hence, the spin-locked reflection phase modulation is achieved using the PB phase under CP wave incidence. In summary, the metasurface meta-atom is realized to switch between anisotropic and isotropic. In addition, the equivalent rotation in the anisotropic state and the capacitance switching in the isotropic state are also achieved.

**Figure 2:**
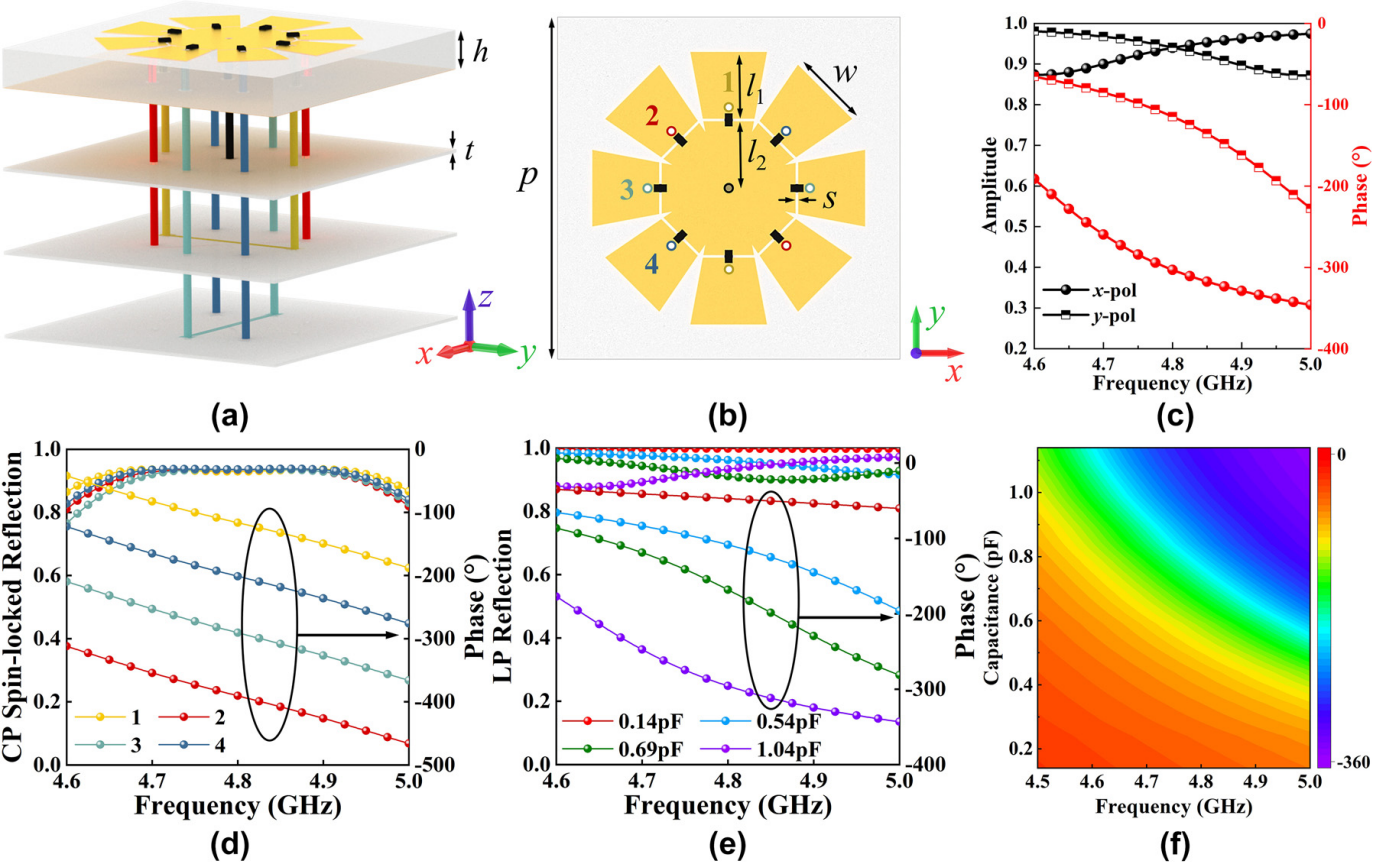
The performance and schematic of the meta-atom. (a) 3D schematic of the proposed meta-atom. (b) Geometric parameters of the proposed meta-atom, where *p* = 20 mm, *l*_1_ = 3.92 mm, *l*_2_ = 3.93 mm, *s* = 0.15 mm, and *w* = 4.5 mm. (c) The reflection amplitude and phase of LP wave incidence when only the varactor on arm 1 is conducted. (d) The amplitude and phase of CP spin-locked reflections when varactor diodes on different arms are turned on (the number 1 in the legend indicates that only the varactor on the arm 1 is conducted, and the other numbers are analogous). (e) The amplitude and phase of LP polarization-locked reflections when changing the capacitance value of the varactors on all arms. (f) The reflection phase varying with frequency and capacitance value of the varactors on all arms.

The meta-atom structure with 8-fold rotational symmetry is designed for multi-bit PB phase operation, as shown in Figure 2a and b. The entire meta-atom structure from top to bottom is a resonant structure, dielectric substrate, metal plate, and three-layer feeding lines. The yellow part is copper, the thickness is 0.017 mm, the conductivity is 5.8 × 10^7^ S/m. The dielectric substrate is Rogers RO4003C with *ε*_*r*_ = 3.55(1 − 0.027*j*). Four identical metal arms form the top resonant structure with an adjacent angle of 45°. Each metal arm has two metal vias (marked by different colors) through the reflective backplate, connected by metal wires on the lower level. The four arms are individually loaded with two varactor diodes, where the two varactor diodes share a positive pole, and the middle metal patch is the negative pole (ground). Here, manipulating the reflection phase of the *x*-polarized wave can be achieved by changing the capacitance of the varactor diodes on arm 3 along the *x*-direction. Similarly, the reflected phase of the *y*-polarized wave can be modulated by changing the capacitance of the varactor diode on arm 1. Here, the capacitance of the diodes on all arms is kept equal, resulting in an isotropic meta-atom so that the reflected phase modulation is the same for *x*-polarized and *y*-polarized wave incidence. Beyond, the resonant frequency increases from 4.5 GHz to 5 GHz as the capacitance value decreases. Therefore, the reflection phase covers 270° at 4.5–5 GHz, caused by the resonant frequency change, as shown in Figure 2f. Here, four typical values are selected as the codes at 4.9 GHz, as shown in Figure 2e. The polarization-locked reflection phase at these four typical values covers 270° while guaranteeing the amplitude greater than 0.9. In fact, the EM wave with arbitrary polarization can be represented as a linear combination of *x*-polarization and *y*-polarization. Hence, the reflection phase of an arbitrarily polarized EM wave can be modulated by simultaneously changing the phase of the *x*-component and the *y*-component via capacitance variation. Therefore, reconfigurable vortex beams can be generated based on the dynamic phase for full polarization.

For CP waves, the capacitance values of the meta-atom in the *x*- and *y*-directions can be independently modulated, thus introducing different phases for *x*- and *y*-components to achieve spin-locked reflections. Here, it is assumed without loss of generality that the RCP wave $\left(\begin{array}{c} 1\\ j \end{array}\right)$ is incident. For example, making the diodes on the arm 1 (*y*-direction) conductive, the capacitance value is 0.14 pF. The diodes on arm 3 (*x*-direction) do not add voltage, and the capacitance value is 1.14 pF. Here, the reflection amplitude and phase at *x*- and *y*-polarized wave incidence in this state are given in Figure 2c. It can be seen that the reflection amplitude is almost equal for *x*- and *y*-polarized waves and greater than 0.8. The reflection phase of the *y*-polarized wave is *π* ahead of the *x*-polarized wave at 4.6–5 GHz. Therefore, the incident CP wave changes from $\left(\begin{array}{c} 1\\ j \end{array}\right)$ to $\left(\begin{array}{c} 1\\ j\cdot e^{j\pi } \end{array}\right)=\left(\begin{array}{c} 1\\ -j \end{array}\right)$ . Meanwhile, the reflected CP wave can be expressed as $\left(\begin{array}{c} -j\\ 1 \end{array}\right)=\left(\begin{array}{c} 1\\ j \end{array}\right)e^{j3\pi /2}$ since the direction of EM wave propagation changes from +*z*-direction to −*z*-direction. Obviously, the polarization of the reflected EM wave is still RCP, realizing spin-locked reflection. Hence, efficient spin-locked reflection is achieved for CP wave incidence when only the varactor diodes on one arm are turned on. Besides, when only the varactor diodes on arm 2 are turned on, the meta-atom structure is equivalent to a *θ* = 45° rotation of the structure when only the varactor diodes on arm 1 are turned on. According to the PB phase, the reflection amplitudes in these two cases are equal, while the phase difference is 2*θ* = 90°. As shown in Figure 2d, the amplitude of spin-locked reflection at 4.6–5 GHz is greater than 0.8, and the phase gradient is strictly 90° at CP wave incidence. Obviously, the manipulation of spin-locked reflection and its reflection phase is realized based on the PB phase under the incident of CP waves. Here, the 2-bit PB phase is achieved by simply conducting the diodes on different arms. Note that the 3-bit PB phase can be easily achieved by increasing the number of metal arms. Overall, the reflection phase of LP and CP waves can be modulated while keeping the polarization of the reflected wave the same as that of the incident wave by independently and temporally modulating the voltage pulse applied to the meta-atom.

## 3 Results and discussion

### 3.1 Full polarization vortex beams generator

**Figure 3:**
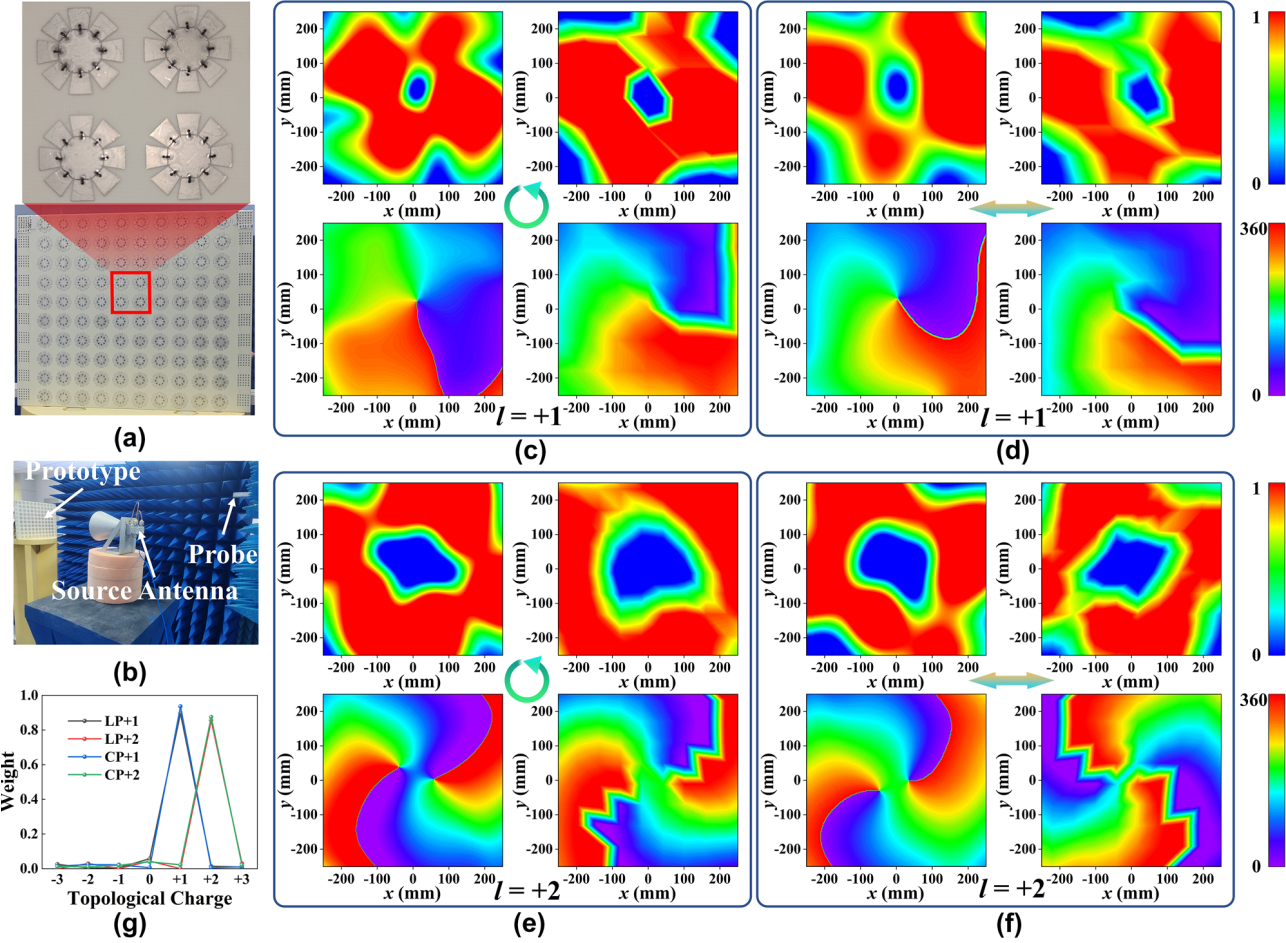
The simulation and measurement results. (a) The front and back of the prototype produced. (b) The measurement setup for the amplitude and phase of the reflected electric field. The simulated (left) and measured (right) amplitude and phase distribution of (c) LCP EM wave with *l* = +1, (d) *x*-polarized EM wave with *l* = +1, (e) LCP wave with *l* = +2, and (f) *x*-polarized EM wave with *l* = +2. (g) The OAM purity of those four cases.

To generate the time-varying vortex beam, an array of 10 × 10 meta-atoms is constructed, and each meta-atom can be fed individually. The specific feeding structure is illustrated in Figure 3a. A prototype was fabricated to exclude the effects of welding and inaccuracy of the varactor diode equivalent circuit model, as shown in Figure 3a. For arbitrary polarization incidence, the reflected electric field of the vortex beams can be represented in the column coordinates be expressed as:

(1) $$E_{r}\left(\rho ,\varphi \right)=E_{0}\left(\rho ,\varphi \right)\exp\left(j\omega t\right)\exp\left(-jl\varphi \right)$$

where *E*_0_(*ρ*, *φ*) is the amplitude, *ρ* is the radiation radius of the reflected EM wave, *φ* is the spatial phase, and *l* is the topological charge number, which can take any integer value. To introduce the additional phase factor exp(*jlφ*), the metasurface array space and reflection phases are discretized into eight different regions *φ*_1_–*φ*_8_. Here, only four independent DC powers are required to realize the full polarization vortex beam generator since only four reflection phases are selected. The phase distribution of topological charges *l* = +1 and *l* = +2 can be realized by four reflection phases. The meta-atom states corresponding to each phase for different polarized EM wave incidence have been given in Figure 2. The simulated and measured results are given only for LCP and *x*-polarized incidence since the meta-atom structure is rotationally symmetric. It should be noted that the signs of the PB phases are exactly opposite for LCP and RCP waves, resulting in a mirror image of the device. Here, it is only needed to mirror the phase distribution to generate vortex beams with the same topological charge for LCP and RCP waves. The simulated electric field amplitude and phase distributions are shown in Figure 3c–f, which indicates that the proposed strategy can generate vortex beams with tunable topological charges under full polarization EM wave incidence. Additionally, the simulation experienced slight distortion, which could be attributed to the following factors: 1) the coupling between the meta-atoms in the metasurface array. 2) The feeding structures of metasurface arrays may impair the performance of vortex beams. 3) The size of the metasurface array is limited. Meanwhile, the fabricated prototype is measured, coinciding with the simulation results. The measurement results are slightly worse than the simulation results mainly due to the following reasons: 1) obstruction of the feed antenna, 2) insufficient near-field scanning accuracy, and 3) fabrication errors. Furthermore, the OAM purity is introduced here:

(2) $$P_{l}=\frac{{\left|A_{l}\right|}^{2}}{\overset{l=+\infty }{\underset{l=-\infty }{\sum }}{\left|A_{l}\right|}^{2}}$$

where *A*_*l*_ is the amplitude of the OAM state with topological charge *l*, which can be represented as:

(3) $$A_{l}=\frac{1}{2\sqrt{\pi }}\underset{xy}{\iint }A\left(x,y\right)\exp\left[j\phi \left(x,y\right)\right]\times \exp\left[-jl\varphi \right]\text{d}x\text{d}y$$

where *A*(*x*, *y*) and *ϕ*(*x*, *y*) are the electric field amplitude and phase sampled along the boundary, respectively. Figure 3g shows that the purity is about 0.9 for generating vortex light carrying *l* = +1 and about 0.8 for vortex light carrying *l* = +2. The purity decreases as the topological charge increases, mainly due to the few bits of the meta-atom. It is also found that the LP wave possesses a higher content of all other order harmonics than the CP wave, except for the +2th harmonic. This phenomenon is primarily due to the unequal amplitudes of the four states of the meta-atom that uphold the *π*/2 phase difference, as shown in Figure 2e. However, the amplitudes of the four states of the meta-atom possess precisely identical amplitudes and exhibit broadband and dispersion-free phase differences for CP wave, as shown in Figure 2d.

### 3.2 Verification of the rotational Doppler effect

The simulations and measurements above show that the proposed strategy can generate vortex beams with tunable topological charges under arbitrary polarized wave incidence. In the following, the rotational Doppler effect and the generation of time-varying vortex beams will be verified by the proposed strategy. When a vortex beam carrying OAM is incident vertically on the surface of a rotating object, its scattered waves generate a related Doppler effect called the rotational Doppler effect [57], [58]. Assuming a vortex beam carrying a topological charge *l* is incident vertically on an object rotating at a speed Ω, the phase change after the rotation angle *θ* through time *t* is [58]:

(4) Δφ/Δt=lΔθ/Δt

Hence, the frequency shift Δ*f* can be represented as:

(5) Δf=lΩ/2π

**Figure 4:**
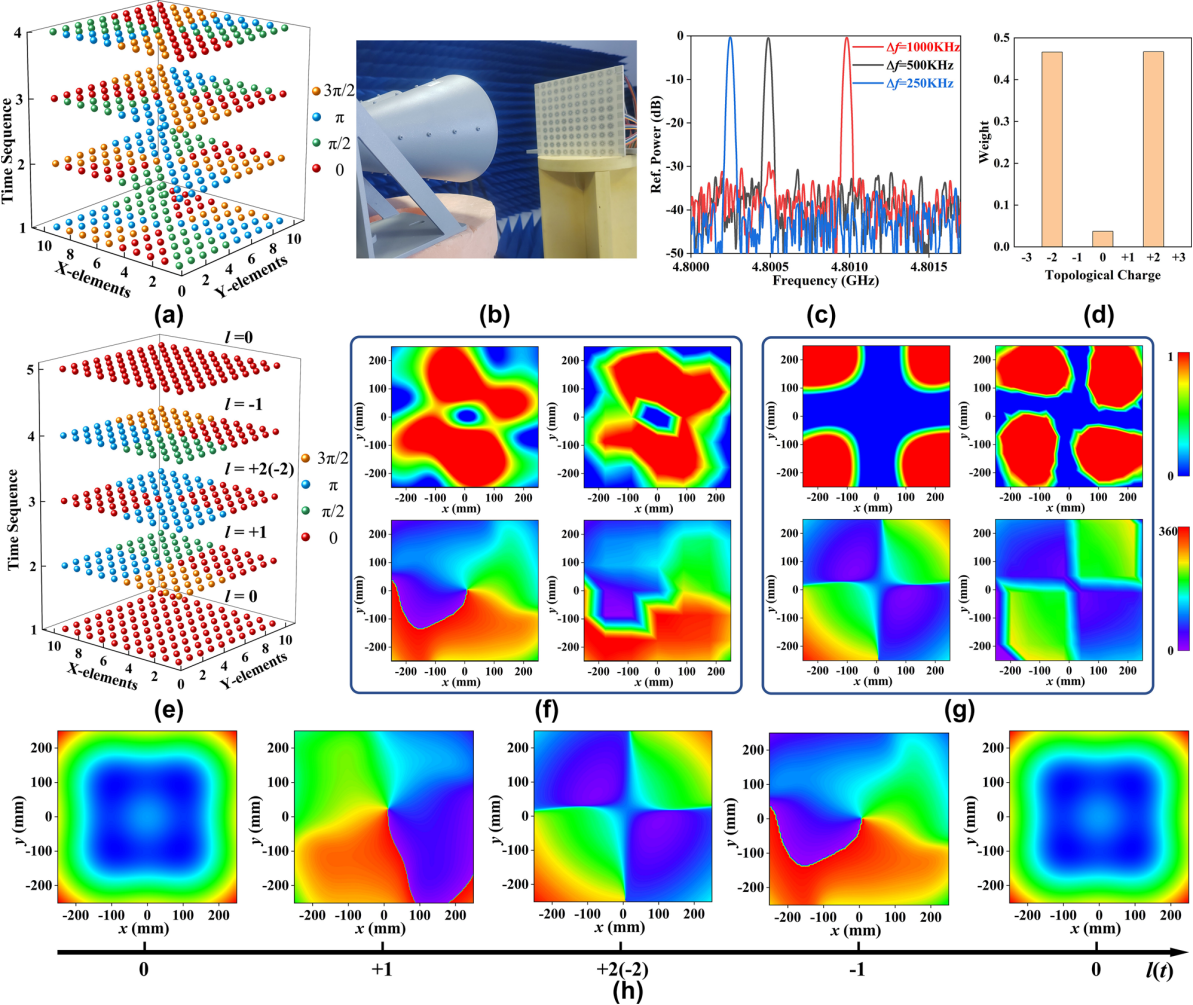
Generation of time-varying vortex light and verification of the rotational Doppler effect. (a) The space-time coding sequences for verification of the rotational Doppler effect. (b) The measurement setup for the rotational Doppler effect. (c) The measured frequency of the reflected EM wave under the codes shown in Figure 4a under the incidence of LCP wave with a frequency of 4.8 GHz. (d) The purity of the generated vortex beam with *l* = +2(−2). (e) The space-time coding sequences for generating time-varying vortex beams. The simulated (left) and measured (right) amplitude and phase distribution of LCP wave with (f) *l* = −1 and (g) *l* = +2(−2). (h) The time-varying phase distributions under the space-time coding sequences shown in Figure 4e.

Here, the metasurface’s equivalent rotation is achieved through adjustment of the voltage distribution rather than mechanical rotation. When the voltage distribution on the metasurface varies periodically with speed *t*_*a*_ according to the space-time coding sequences shown in Figure 4a, the metasurface is equivalently rotating at the speed of Ω = *π*/(4 * *t*_*a*_). The LCP wave with a frequency of 4.8 GHz is chosen as the incident EM wave and generates the vortex beam carrying *l* = +2 as verification. Hence, the frequency shift of the reflected EM wave can be expressed as:

(6) $$\Delta f=1/\left(4*t_{a}\right)$$

Obviously, the frequency shift is only related to the voltage modulation frequency. Therefore, the rotational Doppler effect can be verified by simply measuring the frequency difference between the received and transmitted EM waves. Figure 4c shows the measured spectrum of the reflected EM wave at the modulation speed of *t*_*a*_ = 1 us, 0.5 us, and 0.25 us. The frequency shift is 250 KHz, 500 KHz, and 1000 KHz, which agrees well with the value calculated in Equation (6). The slight offset was due to the purity of the generated vortex beam, synchronization of the four bias voltages, and overcharging of the diode after loading the bias voltage.

### 3.3 Generation of time-varying vortex beams

The phase profile of a time-varying vortex beam is exp(*jl*(*t*)*φ*), where the topological charge varies with time *t*. Thus, the phase of the EM wave varies simultaneously with azimuth and time. Here, only approximately continuous time-varying vortex beams can be generated since the modulation signal can only be periodic, and *l*(*t*) can only take integers ranging from 0, ±1, ±2, etc. Thus, at the *m*-th period, there is

(7) $$l\left(t\right)=\frac{N}{mT}t-\frac{N}{2},\;t=\frac{mT}{N},\frac{2mT}{N},\ldots ,\frac{mT}{2},\ldots ,mT$$

where *N* signifies the number of azimuthal sectors in which the metasurface is segmented, m denotes the number of time periods and takes any positive integer, and *T* is the modulation period. Here, the metasurface is segmented into *N* azimuthal sectors of finite number to realize *N* different OAM profiles. Each sector can only exhibit a specific reflection phase at any given moment. The OAM carried by the vortex beam at the *mT* moment is actually half each of *N*/2 and −*N*/2. Thus, the value of *l*(*t*) is finite, repeating from 1 − *N*/2 to *N*/2 in each period *T*. To demonstrate the described time-varying vortex beams, *N* = 4 is chosen here as a demonstration. Besides, only the fundamental frequency operation is concerned since the modulation period *T* = 10 ms, i.e., the modulation frequency is *f*_*c*_ = 100 Hz, far less than the operating frequency of 4.8 GHz. The phase distribution on the metasurface at different moments is given in Figure 4e, and the corresponding topological charges are taken periodically as *l*(t) = 0, +1, +2(−2), −1, 0. As a demonstration, we measured the amplitude and phase distributions of the electric field at different moments with LCP wave incidence. The amplitude and phase distributions of the measured and simulated electric field at *l* = +1 have been given in Figure 3c. The results for *l* = +2(−2) and *l* = −1 are given in Figure 4f and g, respectively. In addition, the purity of the *l* = +2(−2) was also calculated according to Equation (2), as shown in Figure 4d. The purity of *l* = +2 and *l* = −2 is almost equal and greater than 0.45. In addition, Figure 4h displays the time-varying phase distributions, demonstrating the effectiveness of the proposed strategy in achieving time-varying vortex beams. Furthermore, the expansion of *N* values can be realized by increasing the number of code bits or increasing the size of the array.

## 4 Experimental section

**Simulation:** All simulations were performed in the commercial EM simulation software CST Microwave Studio, based on the finite element method for solving the full 3D Maxwell equations. For the reflection amplitude and phase of the meta-atom, the boundary conditions are periodic in the *x*- and *y*-directions and open (add space) in the *z*-direction, with the LP and CP waves incident along the +*z*-direction. For the simulation of OAM, the boundary conditions are open (add space) in the *x*-direction, *y*-direction, and *z*-direction, and the plane wave is incident along the +*z*-direction.

**Optical Characterization:** All measurements were performed in an anechoic chamber. The measurement setup for the electric field amplitude and phase is given in Figure 3a and b. A near-field experimental system consisting of an experimental bench, motion controller, VNA, scanning probe, and CP (LP) horn antenna was used to measure the electric field distribution. Here, the source antenna is placed at a 400 mm distance from the prototype, and the scanning probe is placed at a 600 mm distance from the prototype to ensure plane wave incidence. The electrical probe is moved in the plane by a robotic arm and records the amplitude and phase distribution data of the *x*-component (E*x*) and *y*-component (E*y*) of the electric field. Then, the total reflection is E_*L*_ = E*x* + iE*y* for LCP wave incidence, and the total reflected right circularly polarized electric field is E_*R*_ = E*x* − iE*y*. The scan range of the electrical probe is 250 mm × 250 mm. Note that the feed antenna is located at the beam null and that the blocking effect is minimized. Each measured near-field pattern consists of 25 × 25 = 625 electric field data with a scanning resolution of 10 mm in both the *x*- and *y*-directions. For measurements of the rotational Doppler effect, the transmit port of Agilent’s vector network analyzer (VNA) E8363B is connected to a C-band CP horn antenna that is used as a transmitter, as shown in Figure 4b. Another CP horn antenna is connected to the spectrum analyzer to detect the frequency change of the received EM wave.

## 5 Conclusions

To summarize, we proposed a strategy for realizing reconfigurable vortex beam generators at arbitrary polarized EM wave incidence. A meta-atom with 8-fold rotational symmetry was designed and loaded with varactor diodes on each symmetry axe. The 2-bit PB phase was realized by conducting the varactor diodes in different directions, and the 2-bit dynamic phase was realized by changing the capacitance of all the varactor diodes. Both simulations and experiments have verified the generation of vortex beams with topological charges of +1 and +2 at the incidence of CP and LP waves, respectively. Furthermore, the time-varying vortex beams were realized, and the rotating Doppler effect was verified by subtly designing the spatio-temporal coding sequence. In addition, the coding bits of the PB phase can be further expanded by increasing the symmetry of the meta-atom, enriching the functionality of the metasurface. This work paves the way for generating vortex beams with reconfigurable topological charge and polarization, which holds potential for information encryption, sensing, and plasma devices.
